# Brazilian joint statement on the management of mechanically ventilated patients: where did we come from? Where should we go?

**DOI:** 10.62675/2965-2774.20250028

**Published:** 2025-04-02

**Authors:** Bruno Adler Maccagnan Pinheiro Besen, João Gabriel Rosa Ramos, Irene Aragão

**Affiliations:** 1 Universidade de São Paulo Faculdade de Medicina São Paulo SP Brazil Postgraduate Programme in Medical Sciences, Faculdade de Medicina, Universidade de São Paulo - São Paulo (SP), Brazil.; 2 Instituto D’Or de Pesquisa e Ensino São Paulo SP Brazil Instituto D’Or de Pesquisa e Ensino - São Paulo (SP), Brazil.; 3 Clínica Florence Salvador BA Brazil Clínica Florence - Salvador (BA), Brazil.; 4 Universidade Federal da Bahia Faculdade de Medicina Salvador BA Brazil Faculdade de Medicina, Universidade Federal da Bahia - Salvador (BA), Brazil.; 5 Centro Hospitalar Universitário do Porto Hospital de Santo Antonio Intensive Care Unit and Emergency Porto Portugual Intensive Care Unit and Emergency, Hospital de Santo Antonio, Centro Hospitalar Universitário do Porto - Porto, Portugual.; 6 Universidade do Porto Instituto de Ciências Biomédicas Abel Salazar Porto Portugal Instituto de Ciências Biomédicas Abel Salazar, Universidade do Porto - Porto, Portugal.

The management of patients undergoing mechanical ventilation (MV) is a core skill for intensivists. Mechanical ventilation is not only a hallmark that has led to what we now know as an intensive care unit, but MV selects the most severely ill cohort of critically ill patients. The development of new technologies, advances in the understanding of physiology and new evidence from clinical trials have highlighted the potential benefits and harms associated with MV. Therefore, guidelines are needed to improve the outcomes of mechanically ventilated patients.

The authors of the *Associação de Medicina Intensiva Brasileira* and the *Sociedade Brasileira de Pneumologia e Tisiologia* now provide an updated Joint Statement on evidence-based practices in MV,^(1)^ which updates a successful document published 10 years ago.^(2,3)^

## Study summary

This Joint Statement is available online (https://indd.adobe.com/view/017f739a-847f-4587-9bef-15b9c01756ba) and provides readers with 100 suggestions and 288 considerations for clinical practice across 38 themes related to mechanically ventilated patients. The statement provides recommendations not only about the process (before-during-after) of MV itself but also about related aspects of the management of mechanically ventilated patients, such as hemodynamic management and multiprofessional team engagement. It is therefore a comprehensive document for critical care clinicians. Its wide scope ensures that these recommendations will be widely used by practitioners who strive to improve their patients’ outcomes.^(1)^

Given its broad scope, this joint statement was not fit to follow the current Grading of Recommendations Assessment, Development and Evaluation (GRADE) recommendations to be called a guideline, as acknowledged by the authors. Nevertheless, the authors designed a process in which pairs of experts from each area developed recommendations, which were validated by a consensus process during a face-to-face meeting organized by the steering committee of the joint statement. The authors used the following two terms: (1) **suggestions**, when recommendations were based on at least one low risk of bias randomized clinical trial or systematic review or consolidated recommendations from international societies; and (2) **considerations**, which could be based on clinical trials with a high risk of bias or observational studies or expert opinions.

## Key caveats and limitations

The most important caveat in using this statement for clinical practice is that although the authors did not use the term "guideline" or GRADE wording for recommendations (i.e., strong recommendations, conditional recommendations and best practice statements), it is very likely that the reader may use this document according to these forms of guidance, as if it were a guideline. In this case, this document brought together a wide-ranging panel of experts, who reflected on ventilation practices and the literature using the terms "suggestions" (for recommendations with stronger evidence) and "considerations" (for recommendations with the least evidence).

Readers should nevertheless be aware that the document did not follow all current recommendations for guideline development: (i) the suggestions and recommendations are not based on an updated systematic review of the best available evidence for each question; (ii) the certainty of evidence was not assessed following the GRADE approach by considering the risk of bias, imprecision, inconsistency, indirectness and publication bias;^(4)^ and (iii) the document did not follow evidence-to-decision frameworks,^(5)^ which allows the reader to understand how each recommendation was arrived at and the weights given to the balance between desirable and undesirable effects, along with resource use, equity, acceptability and feasibility considerations.

Importantly, considering possible inequities and heterogeneous access to care and technology, it would be desirable to understand implementation considerations, as many of the suggestions include interventions that are either controversial—and therefore possibly do not change clinical outcomes—or associated with increased costs to health care systems and not necessarily cost-effective for different willingness-to-pay thresholds.

## Relationships with other guidelines

Table 1 compares recommendations from the Brazilian joint statement on MV to overlapping North American and/or European guidelines.^(6–12)^ We focused this comparison on the following three core aspects of MV: ventilator settings and other therapies for acute respiratory distress syndrome, weaning from the ventilator and noninvasive respiratory strategies.

**Table 1:** Comparison of Brazilian Joint Statement suggestions and considerations to the recommendations of other guidelines regarding noninvasive support, management of ARDS and weaning

Topic	Brazilian Joint Statement	Other guidelines (ERS/ATS/ESICM)
**Noninvasive respiratory support**		
HFNC over COT for hypoxemic respiratory failure	Suggested as a first-line strategy for mild-to-moderate hypoxemia	Conditional recommendation, moderate certainty^(8)^ Strong recommendation, moderate certainty^(7)^ Conditional recommendation, low certainty^(9)^
HFNC over COT for postextubation respiratory failure	Do not consider	Conditional recommendation, low certainty^(9)^
NIV for postextubation respiratory failure	Suggested against (i.e., strong recommendation against)	Conditional recommendation against, low certainty^(11)^
NIV for hypoxemic respiratory failure	Consider	No recommendation because of uncertainty in evidence^(11)^
NIV for hypercapnic respiratory failure	Suggested	Strong recommendation, high certainty^(11)^
NIV for acute cardiogenic pulmonary edema	Suggested	Strong recommendation, moderate certainty^(11)^
**Management of ARDS under mechanical ventilation**		
Lung protective ventilation (4 - 8mL/kg and Pplat < 30cmH_2_O)	Suggested	Strong recommendation, moderate certainty^(5)^ Strong recommendation, high certainty^(7)^
Driving pressure ≤ 15cmH_2_O for all severity categories of ARDS	Consider (i.e., conditional recommendation)	Not addressed elsewhere
Mechanical power	Do not consider	Not addressed elsewhere
Prone positioning for severe ARDS	Suggested	Strong recommendation, moderate certainty^(5)^ Strong recommendation, high certainty^(7)^
Higher PEEP in moderate-severe ARDS	No recommendation made for any specific PEEP titration strategy	Conditional recommendation, moderate certainty^(5)^ No recommendation^(7)^
Prolonged recruitment maneuvers in moderate-severe ARDS	Do not consider	Strong recommendation against, moderate certainty^(6,7)^ Strong recommendation against, moderate certainty^(7)^
VV-ECMO for severe, refractory ARDS	Suggested	Conditional recommendation, low certainty^(6)^ Strong recommendation, moderate certainty^(7)^
ECCO_2_R for ARDS	Do not consider	Strong recommendation against, high certainty^(7)^
Steroids for ARDS	Not addressed	Conditional recommendation, moderate certainty^(6)^
Neuromuscular blockers for early severe ARDS	Consider	Conditional recommendation, low certainty^(6)^ Strong recommendation against routine use, moderate certainty^(7)^
**Weaning**		
Pressure support augmentation during spontaneous breathing trials	Suggested	Conditional recommendation, moderate certainty^(10)^
Sedation minimization	Suggested	Conditional recommendation, low certainty^(10)^
Postextubation NIV for high-risk patients	Suggested	Strong recommendation, moderate certainty^(10)^ Conditional recommendation, low certainty^(11)^
Early mobilization on mechanical ventilation	Consider	Conditional recommendation, low certainty^(10)^
Ventilator liberation protocols	Not explicitly addressed; daily weaning readiness assessment is suggested	Conditional recommendation, low certainty^(10)^
Cuff leak tests	Suggested	Conditional recommendation, very low certainty^(10)^
Systemic steroids for failed cuff leak tests	Suggested	Conditional recommendation, moderate certainty^(10)^

ARDS - acute respiratory distress syndrome; ERS - European Respiratory Society; ATS - American Thoracic Society; ESICM - European Society of Intensive Care Medicine; HFNC - high-flow nasal cannula; COT - conventional oxygen therapy; NIV - noninvasive positive-pressure ventilation; Pplat - plateau pressure; PEEP - positive end-expiratory pressure; VV-ECMO - venovenous extracorporeal membrane oxygenation; ECCO_2_R - extracorporeal CO_2_ removal.

For this comparison, we equated suggestions to strong recommendations and considerations to conditional recommendations. Although we recognize that this may not be a straightforward comparison, as discrepancies are observed in the strength of recommendations, most recommendations are in the same direction, highlighting the usefulness of the document. Where there is more controversy than consensus, readers will benefit from acknowledging this uncertainty, which will foster better decision-making at the bedside.

## Implications for research, policy and practice

In future guideline development efforts, this statement may benefit from more focused guidance on specific aspects of MV, taking into consideration specific needs that would also provide a roadmap for further research focused on Brazil's needs.^(13)^ Although developing an evidence-based guideline from scratch may require considerable investment, the current guideline methodology allows the use of ADOLOPMENT, a more efficient guideline adaptation process for resource-constrained settings when trustworthy guidelines already exist.^(14)^

We emphasize that the interpretation and direct application of any guideline recommendations to institutional policy should always be cautious. Although reduced variability in care is desired, unintended consequences may occur when unduly constraints are imposed on clinical decision-making.^(15)^ Areas of controversy should be acknowledged, and clinical decision-making should be allowed to vary within reasonable grounds when residual evidence uncertainty exists.

For clinical practice, this comprehensive document will continue to be an updated reference for those who wish to learn and improve their knowledge and skills in the management of mechanically ventilated patients.

Ultimately, this document provides a roadmap for Brazilian intensivists and other stakeholders involved in the management of mechanically ventilated patients to improve their clinical outcomes. As for any guideline, this document should be used wisely.
